# The protease DDI2 regulates NRF1 activation in response to cadmium toxicity

**DOI:** 10.1016/j.isci.2022.105227

**Published:** 2022-09-27

**Authors:** Sérgio T. Ribeiro, Aude de Gassart, Sarah Bettigole, Lea Zaffalon, Claire Chavarria, Melanie Op, Kalvin Nugraha, Fabio Martinon

**Affiliations:** 1 Department of Immunobiology, University of Lausanne, 155 Ch. des Boveresses, Epalinges 1066, Switzerland; 2 Volastra Therapeutics, 1361 Amsterdam Avenue, Suite 520, New York, NY 10027, USA

**Keywords:** Biological sciences, Molecular biology, Molecular mechanism of gene regulation

## Abstract

DNA-damage inducible 1 homolog 2 (DDI2) is a protease that activates the transcription factor NRF1. Cellular models have shown that this pathway contributes to cell-stress adaptation, for example, on proteasome inhibition. However, DDI2 physiological function is unknown. Ddi2 Knock-out (KO) mice were embryonic lethal. Therefore, we generated liver-specific Ddi2-KO animals and used comprehensive genetic analysis to identify the molecular pathways regulated by DDI2. Here, we demonstrate that DDI2 contributes to metallothionein (MT) expression in mouse and human hepatocytes at basal and upon cadmium (Cd) exposure. This transcriptional program is dependent on DDI2-mediated NRF1 proteolytic maturation. In contrast, NRF1 homolog NRF2 does not contribute to MT production. Mechanistically, we observed that Cd exposure inhibits proteasome activity, resulting in DDI2-mediated NRF1 proteolytic maturation. In line with these findings, DDI2 deficiency sensitizes cells to Cd toxicity. This study identifies a function for DDI2 that links proteasome homeostasis to heavy metal mediated toxicity.

## Highlights

• DDI2 is required for embryonic development
• DDI2 contributes to MT1 and MT2 expression
• Cadmium mediated proteasome inhibition activates DDI2 mediated NRF1 activity
• DDI2 contributes to cell survival on Cadmium-induced toxicity

## Graphical abstract

**Figure:**
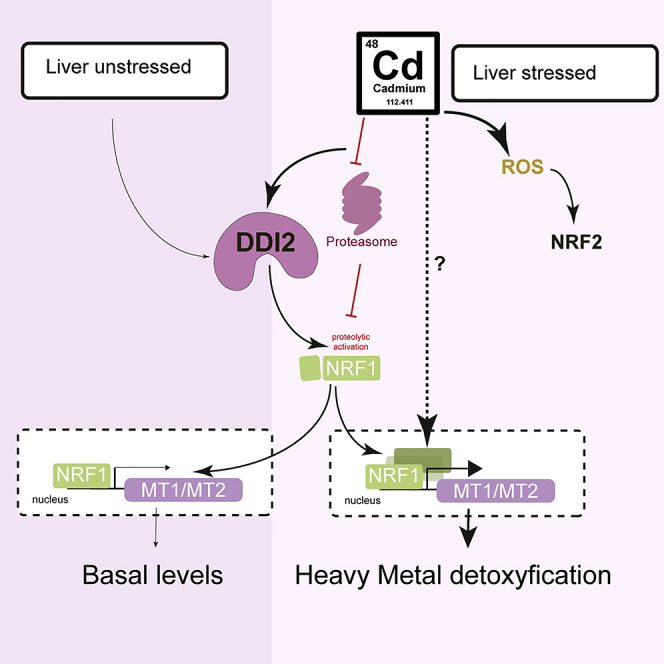

## Introduction

The molecular mechanisms of cell stress response play a central role in cell homeostasis as well as in pathogenesis in different diseases such as cancer and neurodegeneration (Cubillos-Ruiz et al., 2017). The DNA Damage Inducible 1 Homolog 2 (DDI2) is an aspartic protease containing a retroviral protease-like (RVP) domain, highly conserved in eukaryotes (Perteguer et al., 2013; Siva et al., 2016). DDI2 is ubiquitously expressed and implicated in cell stress response. However, DDI2 function, regulation, and downstream molecular mechanisms are largely unknown. Recent data from *Caenorhabditis elegans*, yeast, and human cells demonstrate the involvement of DDI2 in protein homeostasis by regulating proteasome activity (Koizumi et al., 2016; Lehrbach and Ruvkun, 2016), as well as in cell stress response during DNA replication (Kottemann et al., 2018; Serbyn et al., 2020). Koizumi and collaborators reported that DDI2 modulates proteasome subunits through activation of the transcription factor Nuclear factor erythroid-2–related factor 1 (NRF1, encoded by the *NFE2L1* gene) (Koizumi et al., 2016; Rousseau and Bertolotti, 2018). At basal, NRF1 is glycosylated in the ER and translocated to the cytoplasm, where the proteasome continuously degrades it. However, NRF1 accumulates in the cytoplasm on proteasome inhibition and is proteolytically cleaved by DDI2. This process releases an active form of NRF1 that translocates to the nucleus and induces a transcriptional program including the expression of proteasomal (PSM) genes. This adaptation mechanism is known as the proteasome bounce-back response (Koizumi et al., 2016; Radhakrishnan et al., 2010, 2014; Zhang et al., 2007). Consistent with NRF1 contribution to proteasome homeostasis, homozygous deletion of *Nfe2l1* is lethal in mice (Chan et al., 1998) and late-stage deletion of *Nfe2l1* in neuronal cells leads to neurodegeneration (Lee et al., 2011). Although NRF1 target genes are poorly understood, it is described that NRF1 binds the antioxidant response elements (ARE), found in the promoter region of *PSM* and metallothionein (*Mt*) genes. In addition, the depletion of *Nfe2l1* in the mouse liver impairs the expression of *Mt1* and *Mt2* genes (Ohtsuji et al., 2008). MT1/2 are small cysteine-rich and metal-binding proteins, involved in metal-ions chelation and detoxification of Cadmium and Arsenic. (Habeebu et al., 2000; Klaassen et al., 2009; Ohtsuji et al., 2008; Wang et al., 2020; Zhao et al., 2011). Cd is a toxic heavy metal widely present in the environment and exhibits cytotoxic and carcinogenic effects in both mice and humans. It is known that Cd induces the accumulation of ubiquitinated proteins, affects the Ubiquitin Proteasome System (UPS), and causes cytotoxicity and apoptosis in both mouse and human cells (Figueiredo-Pereira et al., 1997; Yu et al., 2011). Moreover, the UPS has been shown to mediate the degradation of abnormal proteins and cell resistance to Cd exposure (Jungmann et al., 1993). Previous studies suggested the use of Cd complexes as proteasome inhibitors that activate cell stress and induce apoptosis in human cancer cells (Yeh et al., 2022; Zhang et al., 2015).

Similar to NRF1, NRF2 is able to bind to ARE regions and mount a cell stress response to promote cell survival. However, the regulatory mechanisms for NRF2 activation, target genes, and its requirement for normal mouse development seem to be distinct from NRF1 (Chan et al., 1996; Ohtsuji et al., 2008).

Here, we found that DDI2 is required for normal embryonic development in mice. The depletion of *Ddi2* in the mouse liver resulted in an impaired NRF1 activation and reduced downstream *MT* gene expression, independently of NRF2. Furthermore, DDI2 appeared to play a central role during metal-based treatments with Cadmium because DDI2-KO cells are more sensitive to cell death compared to WT DDI2 sufficient cells. Overall, we present further understandings into the molecular mechanisms of the DDI2-NRF1-MT pathway during cell stress response in both human cells and mice.

## Results

### DDI2 modulates steady-state metallothionein expression *in vivo*

**Figure 1:**
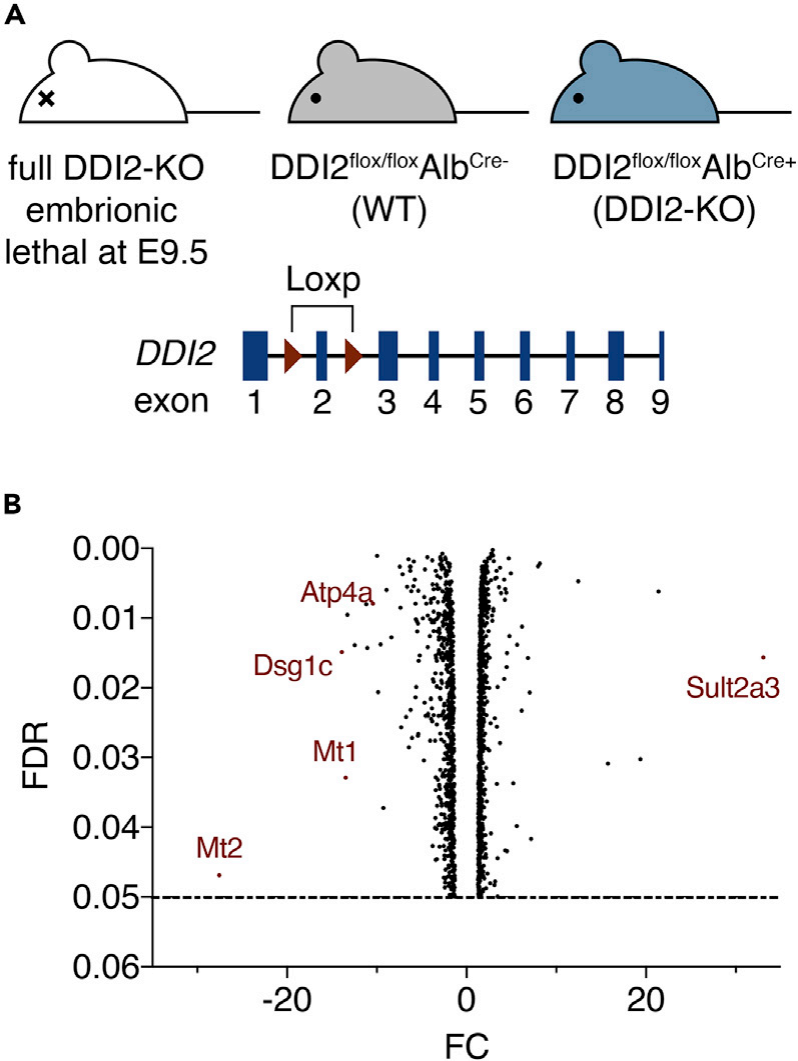
DDI2 modulates metallothionein gene expression *in vivo* (A) Schematic of mouse models: Full DDI2-knockout (KO); wild-type (WT) and liver specific DDI2-KO (up) and representation of *DDI2* locus highlighting the *LoxP* sites used to generate the DDI2^flox/flox^ mice (down). (B) Volcano plot from RNA-sequencing data from liver samples of DDI2-KO compared to WT littermate control mice. The fold change (FC) is plotted on the xaxis, and the false discovery rate (FDR) (adjusted pvalue) is plotted on the yaxis. The represented genes have an FDR lower than 0.05 (horizontal dash line). Some genes are highlighted. The full raw data can be found on the supplementary material. Data points generated from two biological replicates.

**Figure 2:**
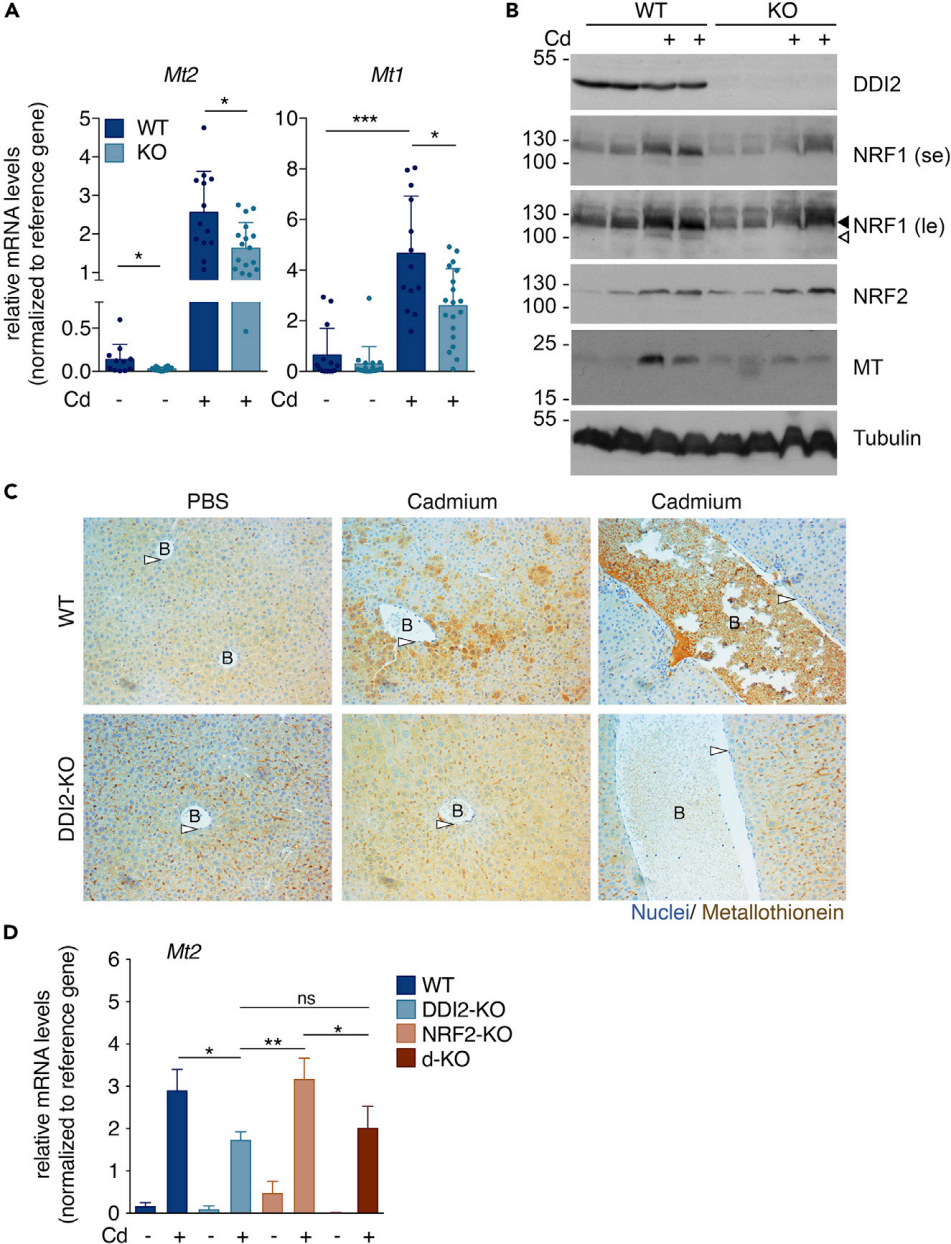
DDI2 activates metallothionein in response to cadmium exposure (A and B) Relative mRNA levels (A) and protein levels (B) from liver samples of DDI2-KO (KO) and wild-type (WT) mice after 16 h of intraperitoneal injection of PBS or 8 mg/kg of CdCl_2_ (Cd). (A) mRNA levels of metallothioneins, *Mt1* and *Mt2*, normalized with *Actin* mRNA levels, each dot represents a single animal. n = 11, 14 for WT and n = 13, 17 for DDI2-KO, treated with PBS or Cd, respectively. (B) Representative immunoblotting (n = 3) showing the relative protein levels of DDI2, NRF1, NRF2, MT and tubulin as loading control, of liver samples as in (A). Each line represents one animal and the protein molecular weights in KDa are indicated. ◀ indicates the full-length protein, ◁ indicates the cleaved protein, se: short image exposure, le: long image exposure. (C) Representative micrographs (n = 3) of immunohistochemistry staining of MT proteins from liver tissues as in (A). (D) mRNA levels of *Mt2*, normalized with *Actin*, from liver samples from WT, DDI2-KO, NRF2-KO, and DDI2- and NRF2- double-KO (d-KO) from animals treated as in (A) (n = 5). p values were calculated using two-tailed unpaired Mann-Whitney t-tests and error bars denote SD. ∗p<0.05; ∗∗p<0.01; ∗∗∗p<0.001; B, blood vessel; arrow, epithelial cells.

The aspartic protease DDI2 is highly conserved between species and ubiquitously expressed; however, its function is poorly understood. DDI2 was recently described to mediate the activation of NRF1 during proteasome inhibition (Koizumi et al., 2016). To further elucidate DDI2 function, we generated DDI2 deficient mice. Full body depletion of DDI2, using gene-trap technology, was embryonic lethal at middle gestational stage with decreased viability observed at E9.5 (Figures 1A and S1), confirming previous observations (Siva et al., 2020). However, a DDI2 liver-specific depletion, using conditional deletion of DDI2-floxed under albumin control was viable and the toxicology results were similar to WT animals at steady state, except for catalase activity, that was reduced (Figure S2A). At non-stressed conditions, DDI2-KO mice presented similar liver tissue structure and morphology compared to the WT animals. To identify DDI2 downstream target genes, we performed mRNA sequencing using liver samples from DDI2-KO mice and littermate controls (Figure 1B). Gene ontology (GO) analysis of molecular function showed that many genes affected by DDI2 deficiency belong to the category of DNA binding and transcriptional regulators (Figure S2B). Several genes involved in the metabolic process (Atp4a, Slc25), cellular response to ions and transcription regulation (Mt1, Mt2) are disturbed in DDI2-deficient livers (Figure 1B and S2B). The top downregulated hits identified in the genetic screening were the metallothionein genes, *Mt1* and *Mt2* (Figure 1B). *MT* genes were previously described to be under the control of the transcription factor NRF1 (Ohtsuji et al., 2008), suggesting that DDI2 may contribute to NRF1 activation at basal in the mouse liver. These findings were confirmed by real-time PCR mRNA expression analysis in the liver of deficient animals (Figure 2A). To analyze stress-induced *Mt* genes expression, we challenged mice with heavy metals. Intraperitoneal injection of acute doses of cadmium (Cd) induced a robust *Mt* gene expression in the liver of WT animals. In contrast, DDI2-KO mice presented a reduced *Mt1* and *Mt2* genes induction after 16 h of Cd treatment (Figure 2A). Importantly, we found that Cd exposure triggered proteolytic activation of NRF1 in the mouse liver (Figure 2B) as well as in human HepG2 cell line (Figure S3A). We observed that NRF1 protein levels increase following Cd treatment because of an impairment in NRF1 protein degradation whereas NRF1 mRNA levels remained unchanged (Figure S3B). Moreover, similar to previous studies in human cell lines treated with proteasome inhibitors (Koizumi et al., 2016), we found that DDI2 depletion in mouse hepatocytes prevented Cd-mediated NRF1 cleavage and activation in the liver (Figure 2B). In addition, we confirmed that, similarly to proteasome inhibitors, treatment with Cd can promote NRF1 cleavage in human HepG2 cells (Figure S3C).

To further analyze the contribution of DDI2 to Cd exposure, we immunohistochemically stained MT proteins in liver tissue sections. We confirmed that MT is increased following 16 h of Cd injection (Figures 2B and2C). The WT mouse showed a high MT protein expression with a heterogeneous pattern throughout the liver (Figure 2C, mid-panel). When we stained a transversal cut of the blood vessel, it was possible to identify the high levels of secreted MT protein (Figure 2C, top right panel). These results confirmed the previous reports suggesting that MT proteins chelate Cd molecules in the liver, the MT-Cd complexes are transported to the bloodstream to be later filtered in the kidneys and eliminated from the body through urine (Sabolic et al., 2010). In contrast, after Cd exposure, DDI2-KO animals expressed a diffused pattern of MT in the liver, and showed less secreted MT into the blood vessels than WT animals (Figure 2C, bottom panel).

In addition to NRF1 activation, we also observed an accumulation of NRF2 protein following Cd treatment, independent of DDI2 (Figure 2B). Our results align with previous reports where NRF2 was described to mediate an adaptive cellular response to Cd-induced oxidative stress (Chen et al., 2021; Chen and Shaikh, 2009). To determine if NRF2 could be involved in MT induction in the absence of DDI2, we generated a double KO mouse (d-KO) with complete deficiency of functional NRF2 and liver-specific DDI2 deletion. Our results indicated that NRF2 is not involved in MT induction following Cd treatment. In the NRF2-KO mouse, we observed MT genes induction following Cd treatment, similar to the WT mouse. In contrast, the double DDI2 and NRF2-KO mice presented a reduced MT response following Cd treatment, identical to that observed in the DDI2-KO mice (Figure 2D). These findings demonstrate the partial but specific contribution of the DDI2-NRF1 pathway to the Cd-mediated MT-gene expression.

### Cadmium induces DDI2-mediated cleavage and nuclear translocation of NRF1 in human HepG2 cells

**Figure 3:**
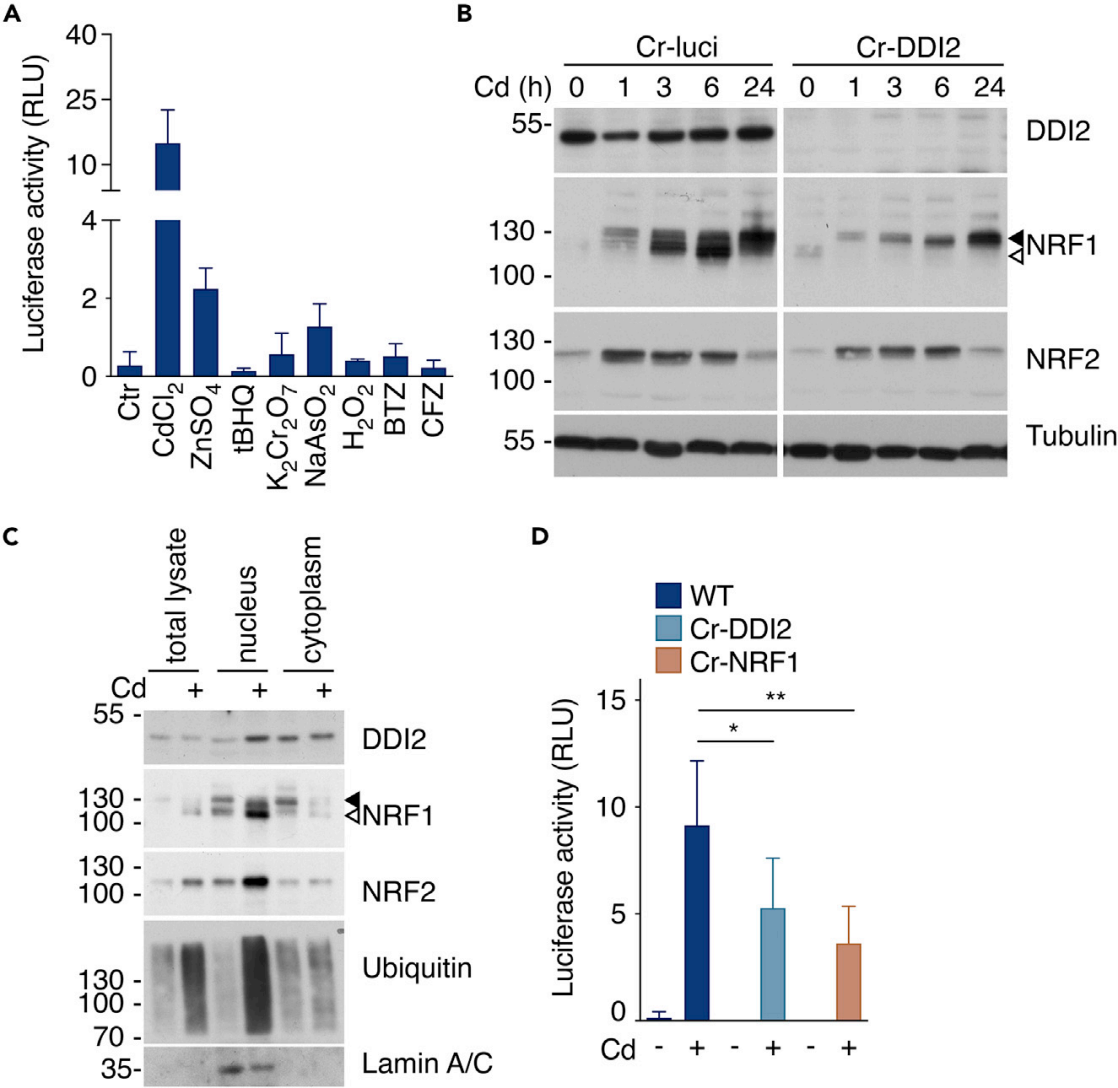
Cadmium induces DDI2-mediated cleavage and nucleus translocation of NRF1 (A) Activation of MT1 promoter-driven luciferase activity in HepG2 cells after 6 h exposure to CdCl_2_ (5 μM), ZnSO_4_ (100 μM), tBHQ (50 μM), K_2_Cr_2_O_7_ (50 μM), NaAsO_2_ (12.5 μM), H_2_O_2_ (1 mM), bortezomib (BTZ, 10 nM), carfilzomib (CFZ, 10 nM) or DMSO as a control (Ctr). (B) Representative immunoblotting (n = 3) showing the relative protein levels of DDI2, NRF1, NRF2 and tubulin as loading control, of CRISPR KO (Cr)-DDI2 or Cr-luciferase (luci) HepG2 cells treated with Cd (5 μM) during the indicated time in hours (h). (C) Representative immunoblotting (n = 3) showing subcellular fractionation of endogenous DDI2, NRF1, NRF2, Ubiquitin and Lamin A/C in total cell lysates, nucleus and cytoplasm from HepG2 cells treated with or without Cd (5 μM) during 6 h. (D) Activation of MT1 promoter-driven luciferase activity in Cr-DDI2, Cr-NRF1 or HepG2 parental cells (WT) after 6 h exposure to Cd (5 μM). Each bar represents the mean, and error bars denote SD of at least three independent experiments. p values were calculated using one-way ANOVA followed by Bonferroni’s multiple comparison test. ∗p<0.05; ∗∗p<0.01. ◀ indicates the full-length protein, ◁ indicates the cleaved protein.

To further confirm the *MT* gene induction following Cd treatment, we used an NRF1-responsive luciferase reporter assay (8xARE-Luc construct characterized in (Ohtsuji et al., 2008)). We transfected the reporter in the human hepatocyte cell line (HepG2). Consistently with MT expression observed in the mouse model, the MT-promoter was activated following Cd treatment (Figure 3A). In addition, in line with previous reports (Chen et al., 2014), Arsenic or Zinc treatments could also increase the MT-promoter activity. In contrast, Bortezomib (BTZ) or Carfilzomib (CFZ), two proteasome inhibitors that promote DDI2-mediated NRF1 activation, could not induce MT-promoter activation *per se*, indicating that Cd, in addition to NRF1, relies on additional factors to trigger MT gene expression. In agreement with our *in-vivo* data where we demonstrate that MT activation is independent of NRF2 activity, the chemical activation of NRF2 with tert-butylhydroquinone (tBHQ) treatment did not induce the MT-promoter activation in HepG2 cells (Figure 3A).

Cd-induced NRF1 accumulation and cleavage is rapid and occurs within hours in a DDI2-dependent manner (Figure 3B). In addition, cell fractionation studies confirmed that proteolytically cleaved NRF1 localized in the nucleus upon Cd treatment whereas uncleaved NRF1 accumulated in the cytoplasm (Figure 3C).

To further confirm the contribution of DDI2 and NRF1 to Cd-mediated *MT* gene expression, we engineered HepG2 with DDI2 and NRF1 deficiency. In agreement with the observations made in DDI2-KO mice, we found that DDI2 or NRF1 deficiency comparably affected Cd-induced MT-promoter activity (Figure 3D). Moreover, NRF1-deficient cells presented undetected levels of MT protein at both basal and Cd-treated conditions (Figure S3A).

### Cadmium inhibits proteasome activity

**Figure 4:**
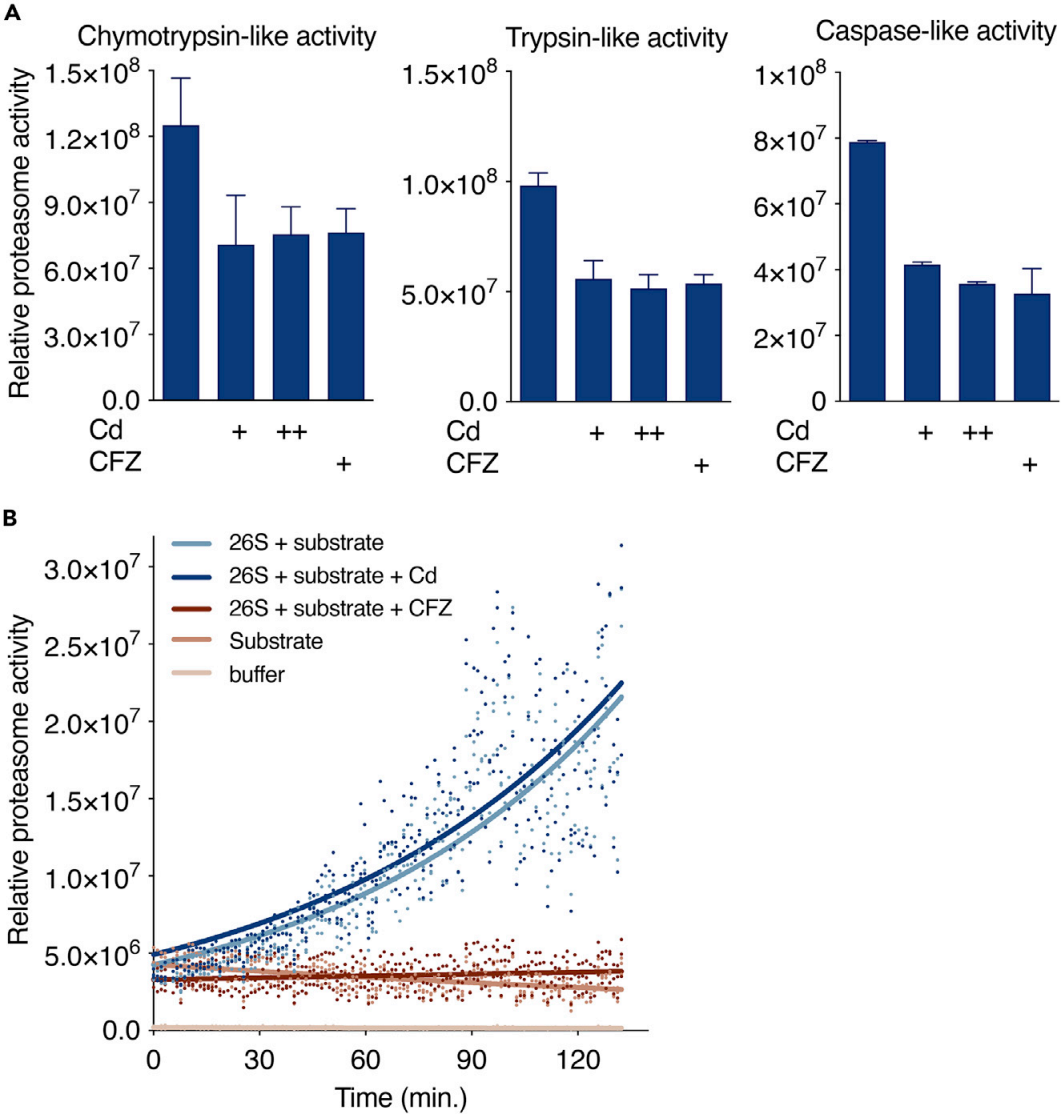
Cadmium indirectly inhibits the proteasome (A) Relative proteasome activity of HepG2 cells treated with CdCl_2_ (Cd; + 1 μM or ++ 5 μM) or carfilzomib (CFZ, 10 nM) during 6 h. Each bar represents the mean, and error bars denote SD of at least three independent experiments. (B) Relative proteasome activity of purified 26S proteasome complexes incubated with the substrate Suc-LLVY-AMC and treated with or without Cd (1 μM) or CFZ (10 nM) during the indicated time in minutes (n = 3).

Although we analyzed NRF1 activation, we observed increased detection of ubiquitinated proteins following Cd treatment (Figure 3C), suggesting that Cd may affect proteasome activity. Because proteasome inhibition can trigger the proteolytic activation of NRF1 (Koizumi et al., 2016; Radhakrishnan et al., 2014), we hypothesized that proteasome impairment could be the upstream mechanism of NRF1 activation following Cd exposure. Surprisingly, we found that Cd treatment in HepG2 cells impaired the proteasome activity, similarly to the proteasome inhibitor, CFZ. The treatment with Cd decreased most proteolytic activities of the proteasome including, chymotrypsin, trypsin, and caspase-like activities (Figure 4A). To determine if Cd directly binds and impairs the proteasome function, we evaluated the proteasome activity using cell-free purified proteasome complexes in the presence of Cd molecules. As expected, CFZ directly interacted with the purified proteasome and blocked the substrate degradation. In contrast, Cd did not inhibit the proteasome in the cell-free assay (Figure 4B), indicating that its mechanism of action is indirect and may affect upstream events required for proteasome function.

Consistent with proteasome inhibition, we observed in mice, that DDI2 deficiency in livers increased K48-linked ubiquitinated proteins (Figure S3D). However, in these conditions we could not observe a robust induction of proteasome genes (Figure S4A). In contrast, treatment with cadmium decreased *PSMA4* mRNA levels in wildtype and NRF2 deficient animals. Because DDI2 deficiency impaired expression of this gene at basal no additional defect was observed on treatment with Cd (Figure S4A). In contrast, in HepG2 cells, cadmium increased PSMA4 gene expression following Cd treatment. As expected, both DDI2- or NRF1-KO cells failed to modulate PSMA4 induction (Figure S4B). These observations indicate that the proteasome bounce back response can be activated but is not always engaged as observed in mice.

### DDI2 contributes to increased cell survival on cadmium-induced toxicity

**Figure 5:**
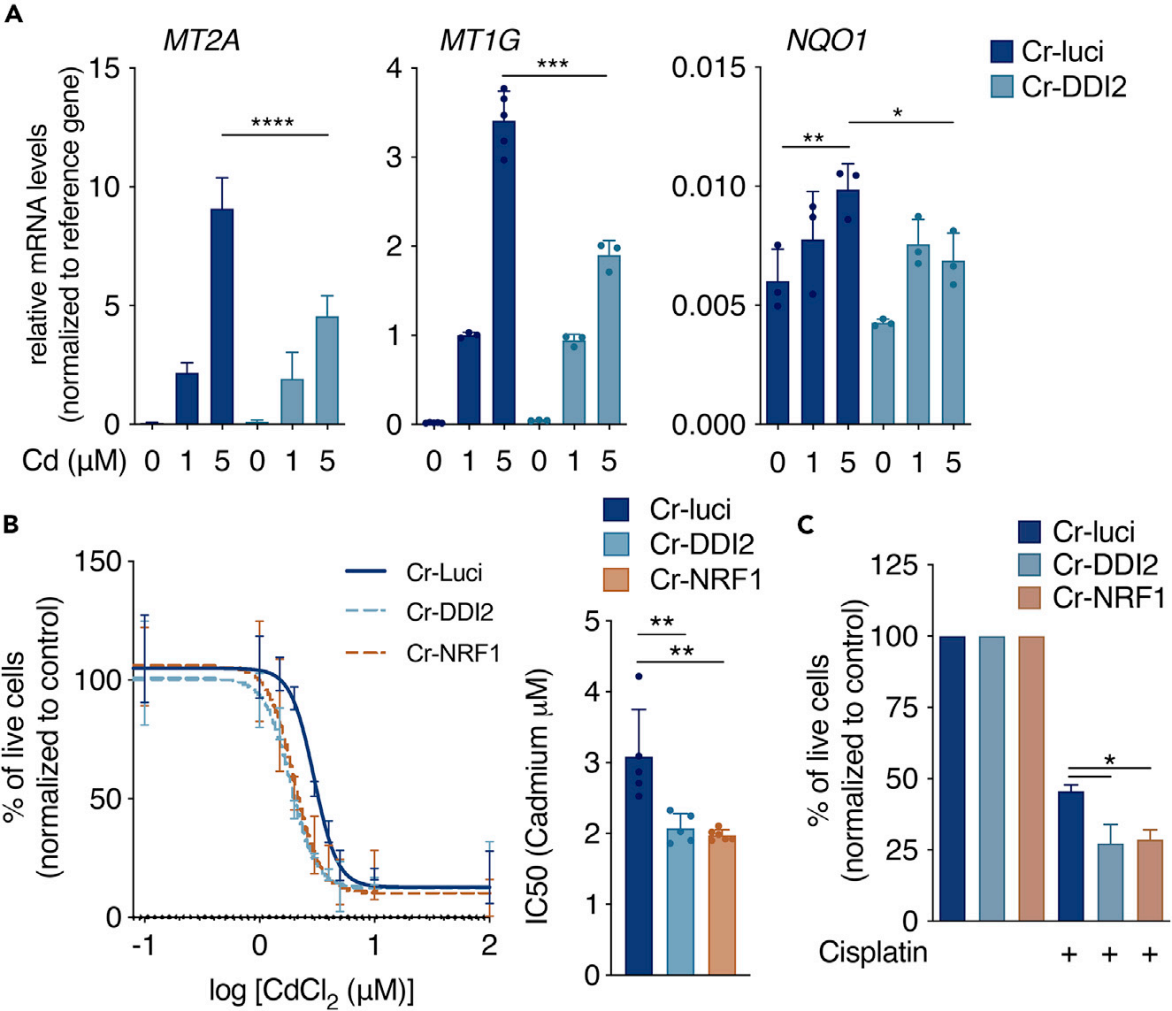
DDI2 promotes cell survival during metal toxicity (A) Relative mRNA levels of *MT2A*, *MT1G* and *NQO1* in CRISPR KO (Cr)-DDI2 or Cr-luciferase (luci) control HepG2 cells treated with CdCl_2_ (Cd, as indicated concentrations) for 6 h. Target genes were normalized with *GAPDH* mRNA levels. (n = 3). (B) Left, Viability of Cr-DDI2 and Cr-luci control HepG2 cells after 48 h treatment with indicated doses of Cd and correspondent IC50 values presented in the bar graph, right (n = 5). Relative viability assessed by 3-(4,5-dimethylthiazol-2-yl)-2,5-dimethyltetrazolium bromide (MTS)/1-methoxyphenazine methosulfate (PMS) assay. (C) Viability of Cr-DDI2, Cr-NRF1 and Cr-luci control HepG2 cells after 48 h treatment with 500 μM of cisplatin, normalized to non-treated cells (n = 3). p values were calculated using two-tailed unpaired Mann-Whitney t-tests and error bars denote SD. ∗p<0.05; ∗∗p<0.01; ∗∗∗p<0.001.

Metallothioneins contribute to the detoxification of heavy metals. We, therefore, tested whether DDI2-mediated *MT* expression could decrease Cd-mediated toxicity. We analyzed *MT* genes expressions in HepG2 cells harboring DDI2 deficiency. In line with the promoter-reporter assays, we observed decreased induction of the human homologs of *MT* genes on treatment with Cd compared with the control population (Figure 5A). We also found that the expression of previously described NRF1 target genes, like *NQO1,* were slightly reduced in Cd-treated HepG2 cells deficient for DDI2, compared to the WT cells (Figure 5A). Moreover, we showed that cellular survival is decreased in DDI2- or NRF1 deficient cells exposed to Cd (Figure 5B), further suggesting that depleting DDI2, consequently preventing NRF1 activation, sensitizes cells to Cd toxicity. Then, we tested if mouse lacking DDI2 in the liver are more sensitive to Cd toxicity. Surprisingly, we did not verify differences in mouse survival, when comparing WT and DDI2-KO, following Cd exposure (Figure S5). However, this observation may be due to the fact that other organs, compensate for MT proteins expression as reported previously (Sabolic et al., 2010), accounting for Cd detoxification and mouse survival.

MT gene and protein levels increase following the exposure of different metals in both mouse and humans. Recently, it has been suggested that MT promotes chemoresistance during metal-based cancer chemotherapies (Borchert et al., 2020; Rodrigo et al., 2021). Cisplatin is a platinum-based compound, commonly used in chemotherapy treatments alone or in combination with other drugs. To test if the DDI2-NRF1-MT axis promotes cell survival and resistance to cancer chemotherapies, we treated HepG2 cell with cisplatin and evaluated the cell survival. Similar to what we observed in cells treated with Cd (Figure 5B), HepG2 cells lacking DDI2 or NRF1 are more sensitive to cell death following cisplatin treatments compared to WT cells (Figure 5C), further indicating that DDI2 and NRF1 can modulate responses to heavy metal exposure.

## Discussion

-This study reports a mouse model to investigate DDI2-associated molecular mechanisms and physiological functions. We used this model to describe the involvement of DDI2 during cell stress response against Cd toxicity. We demonstrated that on Cd exposure in mice, DDI2mediated cleavage and activation of NRF1 and regulated downstream metallothionein (MT) expression in both human and mouse models. Using liver-specific DDI2-KO animals, we found that very few genes were significantly affected by DDI2 deficiency in untreated mice. Unexpectedly, no defects in well-known NRF1 targets such as the proteasome subunits (Koizumi et al., 2016; Lehrbach and Ruvkun, 2016) were identified, except for *Mt1* and *Mt2* expression. These results are consistent with previous reports showing that liver-specific NRF1-KO mice are viable and display reduced expression levels of *Mt1* and *Mt2* (Ohtsuji et al., 2008). These data strongly suggest that DDI2 is upstream of NRF1 signaling pathway in the liver and contributes to *Mt* expression.

Although it has been proposed that both NRF1 and NRF2 share a common target DNA motif, here, we demonstrate that NRF1 controls *Mt* gene expression independently of NRF2. Our observations agree with previous reports showing that both WT and NRF2-KO mice present similar *Mt* expression levels in both Cd-treated and untreated conditions (Chen et al., 2021). However, the activation of MT is not exclusively controlled by the DDI2-NRF1 pathway. *Mt* genes are activated by different response elements, present in the gene promoter, in response to stress stimuli. Previous reports suggest that Cd can displace zinc (Zn) and copper (Cu) from MT conjugates. Consequently, free Zn ions are able to bind and induce the activation of MTF-1 transcriptional factor. Active MTF-1 binds to MRE in the DNA and eventually stimulates the synthesis of *Mt* mRNA (Sabolic et al., 2010). Interestingly, *Mt* null mice are highly sensitive to Cd toxicity (Masters et al., 1994). In comparison, no lethal toxicity was reported in MTF-1-KO mice (Wimmer et al., 2005), and in this study, we did not observe increased lethality in DDI2-KO when treated with 8 mg/kg of Cd. Altogether, *Mt* genes are regulated by distinct mechanisms, including DDI2-NRF1 axis, and are essential to cope with metal toxicity.

The proteasome is a key machinery in eukaryotic cells responsible to cope with cellular toxicity and maintain homeostasis. Importantly, cancer treatments, including proteasome inhibitors, are based on the activation of cell stress to induce apoptosis of targeted tumor cells (Manasanch and Orlowski, 2017). Understanding the molecular mechanisms of cell stress response is crucial to design new therapeutic approaches and to bypass the cancer resistance to treatments. Here, we reported that cellular exposure to metals, as Cd, blocks the proteasome function, increases ubiquitinated proteins, and induces the DDI2-NRF1 stress response pathway to promote the cell survival in hepatocytes. Several studies suggested that metal complexes may regulate proteasome activity (Verani, 2012). However, the molecular mechanisms are largely unknown, these may include the blockade of the UPS by inhibiting de-ubiquitinating enzymes (DUBs) or proteasome assembly chaperones (Chen et al., 2018; Padmanabhan et al., 2016; Xu et al., 2021).

In addition to Cd detoxification, MTs can bind to metal-based drugs, like cisplatin, promoting chemo-resistance in cancer treatments (Borchert et al., 2020; Rodrigo et al., 2021; Si and Lang, 2018). MT expression in tumors is associated with cancer treatment resistance (Si and Lang, 2018) and targeting MT proteins has been suggested to be a powerful tool to boost response rates to metal-based therapies (Borchert et al., 2020). Our data suggest that inhibiting the protease activity of DDI2 could be a suitable therapeutic target to dampen the resistance to metal-based treatments. Nelfinavir is a clinically approved HIV-inhibitor, also described to inhibit DDI2 (Gu et al., 2020). Previous reports demonstrated a beneficial effect of targeting DDI2 using nelfinavir in cancer treatments (Chow et al., 2009) including in Multiple Myeloma (Besse et al., 2018; Driessen et al., 2016; Fassmannova et al., 2020; Gu et al., 2020; Hitz et al., 2019). However, it remains to be evaluated if nelfinavir affects results from inhibition of DDI2-downstream activation of NRF1-MT cellular response.

Altogether, our findings identify heavy metals as activators of the DDI2-NRF1 pathway and downstream MT-chelating proteins. Our results contribute to the understanding of the metal-compounds secretion from the body, as well as the chemotherapy resistance to metal-based treatments.

## Limitation of the study

The contributions of DDI2 during development are still unknown. DDI2-deficient mice die at the mid-gestational stage. Additional studies are necessary to understand the cause of death. Comparing these defects with those observed in NRF1 deficient animals could hint at additional functions for DDI2, possibly independent of NRF1. Although we extrapolate that the phenotypes observed in DDI2 deficiency are mainly a consequence of lack of NRF1 activation, we cannot exclude the contribution of other DDI2 substrates yet to be characterized. For example, NRF3, a close homolog of NRF1, could contribute to DDI2 functions in tissues where it is expressed.

## STAR★Methods

### Key resources table

**Table:** 

REAGENT or RESOURCE	SOURCE	IDENTIFIER
**Antibodies**		

anti-DDI2	This study	N/A
anti-DDI2	Abcam	ab197081
anti-NRF1	Cell signaling	8052
anti-NRF2	Cell signaling	12721, RRID: AB_2715528
anti-Tubulin	Adipogen	AG-27B-0005-C100, RRID: AB_2490494
anti-MT	Enzo Life Sciences	ADI-SPA-550-D, RRID: AB_2039383
anti-ubiquitin	Cell signaling	3936, RRID: AB_331292
anti-K48	Abcam	EP8589
anti-PSMB8	Cell signaling	13726, RRID: AB_2798304
anti-PSMB5	Cell signaling	12919, RRID: AB_2798061

**Chemicals, peptides, and recombinant proteins**		

Bortezomib	LC-Laboratories	B-1408
Carfilzomib	LC-Laboratories	C-3022
H_2_O_2_	Sigma-Aldrich	216763-100ML
CdCl_2_	Sigma-Aldrich	202908-10G
NaAsO_2_	Sigma-Aldrich	35000-1L-R
K_2_Cr_2_O_7_	Sigma-Aldrich	207802-100g
ZnSO_4_	Sigma-Aldrich	83265
tBHQ	Sigma-Aldrich	112941-5g
NaF	Sigma-Aldrich	201154
Na4P2O7	Sigma-Aldrich	P8010
MG132	Sigma-Aldrich	c2211-5mg
Suc-LLVY-AMC	Enzo Life Sciences	BML-P802-0005
Z-ARR-AMC	Calbiochem	CAS 90468-18-1
Z-LLE-AMC	Adipogen	CAS 348086-666-8
AMC	Biovision	K245-100-4
Purified human proteasome complexes	Bio-Techne	E−365-025

**Critical commercial assays**		

RNeasy mini kit	QIAGEN	74104
2X Reverse Transcription master mix	Applied Biosystems	4368814
KAPA HiFi HotStart PCR Kit	KapaBiosystems	KK2502
Pierce BCA Protein assay	Thermo Fisher Scientific	23227
TBARS kit	Abnova	KA4409
Catalase activity	Biovision	K773
SOD activity	Biovision	K335
MTS assay	Promega	G1111
Dual-Luciferase Assay System	Promega	E1910

**Deposited data**		

RNA-seq data	Gene Expression Omnibus	GSE198150
Raw data for immunoblots	Mendeley	https://doi.org/10.17632/9krjdj44p5.1

**Experimental models: Cell lines**		

HepG2	Pascal Schneider, University of Lausanne	N/A

**Experimental models: Organisms/strains**		

DDI2^flox/flox^	This study	N/A
DDI2^flox/flox^Alb^Cre+^	This study	N/A
DDI2^KO^	This study	N/A
NRF2^lacZ/lacZ^	Prof. M. Yamamoto Tohoku University Graduate School of Medicine, Japan	N/A
DDI2^flox/flox^Alb^Cre−^; NRF2^lacZ/lacZ^	This study	N/A

**Oligonucleotides**		

RT-PCR *ACTIN*: fwd 5′-TACCACCA TGTACCCAGGCA-3′ rev 5′-CTCAGGA GGAGCAATGATCTTGAT-3′	This study	N/A
RT-PCR *GAPDH*: fwd 5′-CGCTC TCTGCTCCTCCTG-3′, rev 5′-CGAT GGTGTCTGAGCGAT-3′	This study	N/A
RT-PCR *Mt1*: fwd 5′-ATGGAC CCCAACTGCTCCT-3′, rev 5′-AC AGCCCTGGGCACATTT-3′	This study	N/A
RT-PCR *Mt2*: fwd 5′-CCGATCTC TCGTCGATCTTCAACC-3′, rev 5′-CA GGAGCAGCAGCTTTTCTTGCAG-3′	This study	N/A
RT-PCR *NFE2L1*: fwd 5′-TGGAACAGC AGTGGCAAGATCTCA-3′, rev 5′-GGCA CTGTACAGGATTTCACTTGC-3′	This study	N/A
RT-PCR *NFE2L2*: fwd 5′-TTCCCGG TCACATCGAGAG-3′, rev 5′-TCC TGTTGCATACCGTCTAAATC-3′	This study	N/A
RT-PCR *MT2A*: fwd 5′-CTCTTC AGCTCGCCATGGAT-3′, rev 5′-TG GAAGTCGCGTTCTTTACA-3′	This study	N/A
RT-PCR *MT1G*: fwd 5′-TTGCAA TGGACCCCAACT-3′, rev 5′-TC CTGGATTTTACGGGTCAC-3′	This study	N/A
RT-PCR *NQO1*: fwd 5′-CAGCT CACCGAGAGCCTAGT-3′, rev 5′-AG TGCTCTTCTGCCGACCAT-3′	This study	N/A
RT-PCR *PSMA4* (human): fwd 5′- CCC TTTGGTGTTTTGCT-3′, rev 5’ – GCT GCAGCGCTATTATTTCC-3′	This study	N/A
RT-PCR *Psma4* (mouse): fwd 5’ – AAAAG TGGAAATCGCCACAC-3′, rev 5’ – TTT CTTTCTTCTCCCGCTCA-3′	This study	N/A
SgLuci fwd 5′-CACCGCTTCGAAA TGTCCGTTCGGT-3′, rev 5′- AAAC ACCGAACGGACATTTCGAAGC-3′	(Op et al., 2022)	N/A
SgDDI2 fwd 5′- CACCGGCTCGAAG TCGGCGTCGAC-3′, rev 5′- AAACG GTCGACGCCGACTTCGAGCC-3′	(Op et al., 2022)	N/A
SgNRF1 fwd 5′- CACCGCTTTCT CGCACCCCGTTGTC-3′, rev 5′- AAA CGACAACGGGGTGCGAGAAAGC-3′	(Op et al., 2022)	N/A

**Recombinant DNA**		

LentiCRISPR-v2 vector	Addgene	52961
MT-Firefly-luciferase reporter	Prof. M. Yamamoto Tohoku University Graduate School of Medicine, Japan	(Ohtsuji et al., 2008)

Software and Algorithms		

R bioconductor package “limma”	Bioconductor	R version 3.1.1, limma version 3.20.8

### Resource availability

#### Lead contact

Any additional information or inquiries regarding code availability or resources should be directed to Fabio Martinon (Fabio.Martinon@unil.ch).

#### Materials availability

Request for generated mice models and constructs should be directed to Fabio Martinon (Fabio.Martinon@unil.ch).

### Experimental model and subject details

#### Cell culture and reagents

The human hepatocellular carcinoma HepG2 cell line (originating from a hepatocellular carcinoma of a 15-year-old, white male with liver cancer) was provided from Pascal Schneider (University of Lausanne), and maintained in Dulbecco’s modified Eagle’s medium (DMEM) supplemented with 10% (v/v) fetal bovine serum, 1x non-essential amino acids solution, and cultured in a 37°C incubator with 5% CO_2_. Bortezomib and carfilzomib were purchased from LC-Laboratories (Woburn, MA, USA), cisplatin, CdCl_2_, ZnSO_4_, tBHQ, K_2_Cr_2_O_7_, NaAsO_2_ and H_2_O_2_ from Sigma-Aldrich (St. Louis, MO, USA).

#### Animal models

Animal experiments were performed in accordance with the Swiss animal welfare law and were approved by the local authorities and the animal ethics committee (license numbers: 2390.1; 2390.1a; 2390x2). Both male and female mice, within 6–12 weeks old, were randomly assigned to each experimental condition.

DDI2^flox/flox^Alb^Cre+^ (DDI2-KO) mouse model was generated by microinjection of C57BL/6N-Atm1Brd blastocysts with mouse embryonic stem (ES) cells containing the L1L2_Bact_P cassette (Ddi2^tm1a(EUCOMM)Hmgu^, MGI ID: 4842030) inserted at position GRCm38:Chr4:141,410,874-141450730, following standard methodology. DDI2 full KO mouse was generated by blastocyst microinjection of ES cells containing the pGT1Lxf gene-trap vector (BayGenomics, CA, USA) targeting the *Ddi2* gene (GRCm39:Chr4:141,435,521-141435739). B6J.129P2-Nfe2I2^tmLacz^ (NRF2-KO) mouse were obtained from Prof. M. Yamamoto (Tohoku University Graduate School of Medicine, Japan) (Itoh et al., 1997). DDI2-NRF2 double KO animals and littermate controls were generated by breeding heterozygous DDI2-KO and NRF2-KO mice (DDI2^flox/flox^Alb^Cre+^; NRF2^wt/lacZ^ x DDI2^flox/flox^Alb^Cre−^; NRF2 ^lacZ/lacZ^) (d-KO). Animal genotyping by PCR was done before and after each experiment. All mouse experiments were performed using littermate controls. Both male and female mice were randomly assigned to each experimental condition.

The following oligos were used for genotyping. CRE alleles: CRE frw 5′-AACATGCTTCATCGTCGG-3′ CRE rev 5′-TTCGGATCATCAGCTACACC-3′; Positive band: ∼350 bp. DDI2 flox alleles: frw 5′-GTAACGCCTGGGTCAGGATT-3′ and rev 5′-CCCACAGCCAAGTAAGGAGA-3′; on a 1.5% Gel, WT allele: 188 bp, *LoxP* allele: 268 bp. DDI2 KO alleles: frw 5′-GACAGTATCGGCCTCAGGAAGATCG-3', and rev 5′-TGACTTAGACAGACACTGAG-3′; on a 1.5%, WT band: ∼ 0.5Kb, DDI2 KO band: ∼1.1Kb.

### Method details

#### RNA sequencing

Following total RNA was extracted using RNeasy mini kit (QIAGEN, Hilden, Germany) high-throughput sequencing was performed at the Lausanne Genomics Technologies Facility (University of Lausanne) with Illumina HiSeq 2500 (San Diego, CA, USA) using TruSeq SBS Kit v3 reagents. For the RNA-seq analysis, we used a moderated t-test from the R bioconductor package “limma” (R version 3.1.1, limma version 3.20.8). The data discussed in this publication have been deposited in NCBI’s Gene Expression Omnibus and are accessible through GEO Series accession numberGSE198150.

#### Quantitative real-time PCR

Total RNA was isolated from the same number of cells or similar tissue weight between experimental conditions. RNA was isolated with Trizol (Invitrogen, Waltham, MA, USA) and cDNA was synthesized from 2 μg of RNA using 2X Reverse Transcription master mix (Applied Biosystems, Waltham, MA, USA) following the manufacture’s protocol. The cDNA was quantified by real-time PCR with KAPA HiFi HotStart PCR Kit (Kapa Biosystems, Wilmington, MA, USA) using the LightCycler 480 System (Roche, Basel, Switzerland). Primer sequences used were as follows: *ACTIN*: fwd 5′-TACCACCATGTACCCAGGCA-3′, rev 5′-CTCAGGAGGAGCAATGATCTTGAT-3′; *GAPDH*: fwd 5′-CGCTCTCTGCTCCTCCTG-3′, rev 5′-CGATGGTGTCTGAGCGAT-3′; *Mt1*: fwd 5′-ATGGACCCCAACTGCTCCT-3′, rev 5′-ACAGCCCTGGGCACATTT-3′; *Mt2*: fwd 5′-CCGATCTCTCGTCGATCTTCAACC-3′, rev 5′-CAGGAGCAGCAGCTTTTCTTGCAG-3′; *NFE2L1*: fwd 5′-TGGAACAGCAGTGGCAAGATCTCA-3′, rev 5′-GGCACTGTACAGGATTTCACTTGC-3′; *NFE2L2*: fwd 5′-TTCCCGGTCACATCGAGAG-3′, rev 5′-TCCTGTTGCATACCGTCTAAATC-3′; *MT2A*: fwd 5′-CTCTTCAGCTCGCCATGGAT-3′, rev 5′-TGGAAGTCGCGTTCTTTACA-3′; *MT1G*: fwd 5′-TTGCAATGGACCCCAACT-3′, rev 5′-TCCTGGATTTTACGGGTCAC-3′; *NQO1*: fwd 5′-CAGCTCACCGAGAGCCTAGT-3′, rev 5′-AGTGCTCTTCTGCCGACCAT-3′.

#### Immunoblotting

Cells and tissue protein extracts were prepared with ice-cold RIPA buffer (50 mM NaCl, 50 mM Tris pH 7.4, 1 mM EDTA, 0.1% SDS, 1% NP-40, 1% sodium deoxycholate) supplemented with protease inhibitor cocktail (Roche, Basel, Switzerland), 10 mM Na3VO4, 50 mM NaF, 10 mM Na4P2O7, and 5 μM MG132 (Sigma-Aldrich, St. Louis, MO, USA). Liver tissue was dissociated for 5 min using the Qiagen TissueLyser II (Hilden, Germany). Total protein was quantified by Pierce BCA Protein assay (Thermo Fisher Scientific, Waltham, MA, USA), denaturated and equal amounts were loaded in SDS-PAGE. The following antibodies were used for immunoblot analysis: anti-DDI2 (from our lab, produced from human DDI2 immunized-rabbit serum using HiTrap NHS activated HP columns, GE Healthcare, Chicago, IL, USA), anti-NRF1 (8052) and anti-NRF2 (12,721) from Cell Signaling (Danvers, MA, USA), anti-Tubulin (AG-27B-0005-C100) from AdipoGen (Epalinges, Switzerland), anti-MT (ADI-SPA-550-D) from Enzo Life Sciences (Farmingdale, NY, UA).

#### Plasmid construction and cell line generation

Gene knockout (KO) cell lines were generated by viral transfection of LentiCRISPR-v2 vector (Addgene reference: 52,961) containing the target single guide RNA (sgRNA) sequence as described in (Op et al., 2022). The sgRNA sequence of the Luciferase gene was used as control and denominated as Cr-luci cell line. KO cell lines were selected with 1.5 μg/mL of puromycin (Sigma-Aldrich, St. Louis, MO, USA) for 15 days.

#### Immunohistochemistry

Hematoxylin–eosin (H&E) staining of the liver was performed by the Mouse Pathology Facility (University of Lausanne) according to standard protocols. Briefly, paraffin-embedded liver slides, with 3 μm thick, were treated with Tris-EDTA (pH 9) in a pressure cooker for 2 min. The staining was performed with anti-MT (1/100 in PBS 0.1% BSA, 60 min), Dako EnVision+, and HRP anti-mouse antibody (30 min). The slides were reveled with DAB and counterstain with Harris hematoxylin following standard procedures.

#### Proteasome activity assay

Two million cells were lysed in 300 μL proteasome lysis buffer (50 mM HEPES pH 7.8, 10 mM NaCl, 1.5 mM MgCl_2_, 1 mM EDTA, 1 mM EGTA, 250 mM sucrose and 5 mM DTT in PBS without Ca^2+^ or Mg^2+^). Cell lysates were sonicated 3 s using the microtip output set on ∼3 and centrifuged at 16,000 RCF for 10 min at 4°C. The total proteins were quantified from the supernatant. Equal amounts of total protein were diluted in proteasome lysis buffer supplemented with 2 mM ATP (Sigma-Aldrich, St. Louis, MO, USA) and incubated with 100 μM of proteasome substrate reporters. The following chymotrypsin-, trypsin- and caspase-like substrate reporters were used: Suc-LLVY-AMC from Enzo Life Sciences (#BML-P802-0005, Farmingdale, NY, USA); Z-ARR-AMC from Calbiochem (#CAS 90468-18-1, San Diego, CA, USA); and Z-LLE-AMC from AdipoGen (#CAS 348086-666-8, Epalinges, Switzerland). Fluorescence, A_360_ex/A_460_em, was measured during 60 min at 37°C with SpectraMax i3 (San Jose, CA, USA). AMC (#K245-100-4, BioVision Milpitas, CA, USA) was used as technical positive control. Purified human proteasome complexes (#E−365-025, Bio-Techne, MN, USA) were used for the cell-free proteasome activity assays.

#### Enzymatic assays

Freshly isolated liver tissue was analyzed following the manufacturer’s instructions to quantify the TBARS levels (from 25 mg of tissue, #KA4409, Abnova, Taipei, Taiwan), Catalase activity (from 100 mg of tissue, #K773, BioVision, Milpitas, CA, USA), and SOD activity (from 50 mg of tissue, #K335, BioVision, Milpitas, CA, USA). Blood was collected from the left ventricle and serum was analyzed for ALT, AST and LDH levels using Cobas C111 (Roche, Basel, Switzerland) according to the manufacturer’s instructions.

#### Cytotoxic assay

Cell viability was evaluated by using a 3-(4,5-dimethylthiazol-2-yl)-5-(3-carboxymethoxyphenyl)-2-(4-sulfonphenyl)-2H-tetrazolium (MTS) assay (Promega, Madison, WI, USA) and calculated as the percentage of vehicle control cells. Dose-response curves were plotted using a four-parameter logistic equation. Graphs and IC50 values (the 50% maximal inhibitory concentration) were obtained using GraphPad Prism 9.0 (GraphPad, San Diego, CA, USA).

#### Luciferase assay

For each condition, one million cells were transfected with 0.9 μg MT-Firefly-luciferase reporter plasmid (Ohtsuji et al., 2008) provided by Masayuki Yamamoto (Tohoku University Graduate School of Medicine); 0.1 μg Renilla plasmid was used as the internal control. Dual-Luciferase Assay System (Promega, Madison, MI, USA) was performed 48 h after transfection, according to the manufacturer’s instructions. Specific Firefly luciferase activity was calculated from light intensity measurements and normalized against Renilla luciferase activity as internal control.

### Quantification and statistical analysis

Representative results from at least three independent experiments were shown. Statistical comparisons were made as described in the Figure legends with GraphPad Prism. The significance of the difference was set at p values <0.05. ∗ denotes p<0.05; ∗∗p<0.01; ∗∗∗p<0.001. n indicates the number of independent experiment, except in Figures 2A, 2D, and S5 where it indicates number of animals per group and Figure S1, where N indicates total number of animals.

## Data Availability

The data presented in this publication have been deposited in NCBI’s Gene Expression Omnibus and are accessible through GEO Series accession number: GSE198150. Raw data for immunoblots were deposited on Mendeley Data: https://doi.org/10.17632/9krjdj44p5.1 This paper article not report original code. Any additional information required to reanalyze the data reported in this article is available from the lead contact on request.
