# Supplementary material for: The protease DDI2 regulates NRF1 activation in response to cadmium toxicity
Source: iScience. 2022 Sep 27;25(10):105227. doi: 10.1016/j.isci.2022.105227 (PMC9557025; doi:10.1016/j.isci.2022.105227)
Supplement: Document S1. Figures S1–S5 [file mmc1.pdf]

iScience, Volume *25*

Supplemental information

The protease DDI2 regulates NRF1 activation in response to cadmium toxicity

Sérgio T. Ribeiro, Aude de Gassart, Sarah Bettigole, Lea Zaffalon, Claire Chavarria, Melanie Op, Kalvin Nugraha, and Fabio Martinon

Supplemental figures

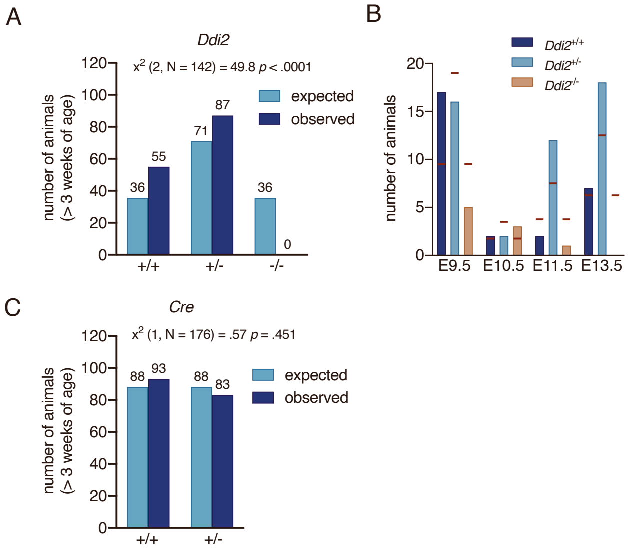

Figure S1. Observed progeny of DDI2 deficient mice, related to Figure 1A

Analysis of all progeny derived from mating heterozygous Ddi2+/- mice, with more than 3 weeks of age (A) or during embryonic development (B). Analysis of all progeny derived from mating Ddi2flox/floxAlbCre-/- and Ddi2flox/floxAlbCre+/- mice, with more than 3 weeks of age (C).

Number of animals per group indicated above the bars, red horizontal lines indicates the number of expected animals. $\chi 2$-test analysis are represented.

A

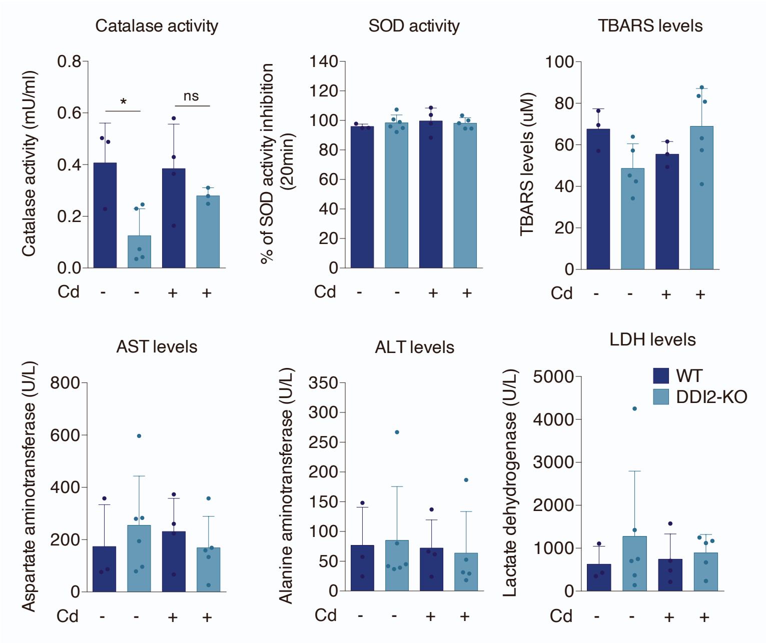

B

Gene Ontology: Biological Process				
#term ID	term description	genes mapped	enrichment score	false discovery rate
GO:0071280	Cellular response to copper ion	4	3.88964	0.0077
GO:0055086	Nucleobase-containing small molecule metabolic proces	53	0.460364	0.0077
GO:0019637	Organophosphate metabolic process	74	0.408501	0.0026
GO:0008610	Lipid biosynthetic process	43	0.404631	0.0076
GO:1901135	Carbohydrate derivative metabolic process	77	0.375283	0.0068
GO:0044281	Small molecule metabolic process	165	0.332546	8.58e-05
GO:1901566	Organonitrogen compound biosynthetic process	130	0.328815	0.0018
GO:0045944	Positive regulation of transcription by rna polymerase ii	102	0.300128	0.0077
GO:0055114	Oxidation-reduction process	130	0.287102	0.00070
GO:0006357	Regulation of transcription by rna polymerase ii	172	0.231131	0.0072

Gene Ontology: Molecular Function				
#term ID	term description	genes mapped	enrichment score	false discovery rate
GO:0003677	DNA binding	162	0.280794	0.00026
GO:0140110	Transcription regulator activity	115	0.262335	0.0058

Figure S2. Impact of DDI2 on liver physiology and gene expression, related to Figure 1B

(**A**) Catalase and superoxide dismutase (SOD) activities and thiobarbituric acid reactive substance (TBARS) levels measured form liver samples; Aspartate aminotransferase (AST), alanine aminotransferase (ALT) and lactate dehydrogenase (LDH) levels measured from serum of wild-type (WT) and liver-specific DDI2-knockout (KO) animals, after 16 h of intraperitoneal injection of PBS or 8 mg/kg of $CdCl_2$ (Cd). Each dot represents a single animal. *p*-values were calculated using two-tailed unpaired Mann-Whitney t-tests and error bars denote standard deviation (SD). *$p<0.05$. (**B**) Gene ontology (GO) analysis of biological process and molecular function generated with RNA-seq data (Figure 1). Functional enrichment analysis obtained with the online software: STING database version 11.5

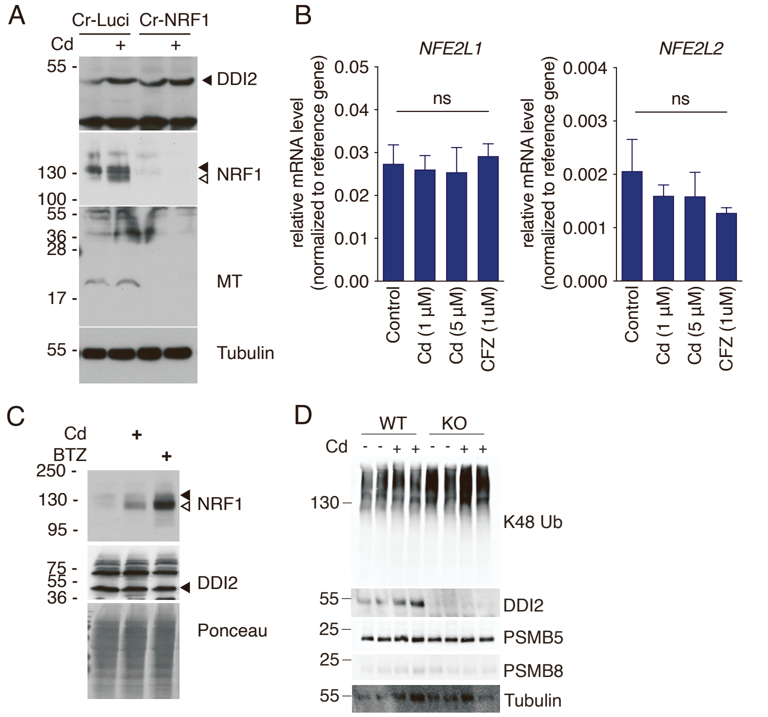

Figure S3 Cadmium promotes NRF1 proteolytic maturation, related to Figure 3

(**A**) Immunoblotting showing the relative protein levels of DDI2, NRF1, ponceau staining, of HepG2 cells treated with cadmium (Cd, 5 μM) or bortezomib (BTZ, 10 nM) for 6 hours (representative of n = 2). (**B**) Relative mRNA levels of NRF1 and NRF2 genes (*NFE2L1* and *NFE2L2*, respectively) normalized with *GAPDH* mRNA levels, from HepG2 cells treated 6 hours with indicated concentrations of cadmium (Cd) or carfizomib (CFZ). (**C**) Immunoblotting showing the relative protein levels of DDI2, NRF1, MT, and Tubulin, of CRISPR KO (Cr)-NRF1 or Cr-luciferase (luci) HepG2 cells treated with or without cadmium (Cd, 5 μM) for 6 hours (representative of n = 2). (**D**) Immunoblotting from liver samples of DDI2-KO (KO) and wild-type (WT) mice after 16 h of intraperitoneal injection of PBS or 8 mg/kg of CdCl2 (Cd) showing the relative protein levels of Ubiquitin-K48, DDI2, PSMB5, PSMB6 and Tubulin as loading control. Each line represents one animal and the protein molecular weights in KDa are indicated. ◀ indicates the full-length protein, ◁ indicates the cleaved protein. Samples were analyzed by two-tailed unpaired Mann-Whitney t-tests and error bars denote standard deviation (SD), ns: not significant.

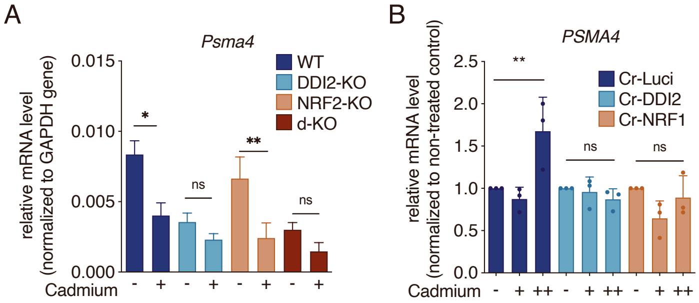

Figure S4. Impact of Cadmium on proteasome subunit expression, related to Figure 4

Relative mRNA levels of *PSMA4* from both mouse models (**A**) or HepG2 cells (**B**). (**A**) liver samples of DDI2-KO (KO), NRF2-KO, double-DDI2-NRF2-KO (d-KO) and wild-type (WT) collected from mice after 16 h of intraperitoneal injection of PBS (-) or 8 mg/kg of $CdCl_2$ (+). (**B**) CRISPR KO (Cr)-DDI2, Cr-NRF1 or Cr-luciferase (Cr-luci) control HepG2 cells treated with $CdCl_2$ (-: 0 µg; +: 1µM, ++: 5 µM) for 6 h. Target genes were normalized with *GAPDH* mRNA levels. (n = 3).

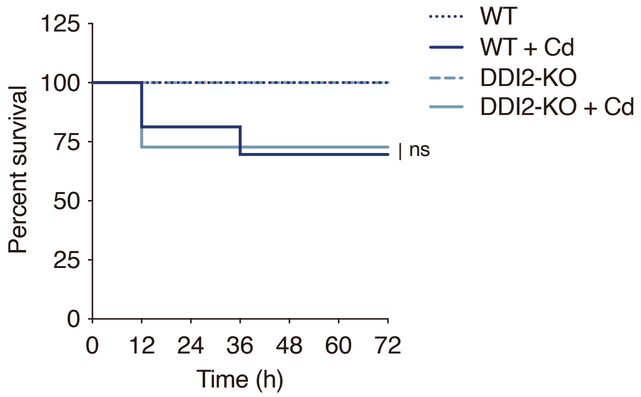

Figure S5. Viability of mice treated with Cadmium, related to figure 5

Percent of survival of DDI2-KO (KO) and wild-type (WT) mice treated with a single dose of cadmium 8 mg/kg (Cd) (n = 15 and 9, respectively) or PBS (n = 12 per group). Survival plots were constructed using Prism software (GraphPad Software Inc., version 9.1.2) with corresponding evaluation using two-sample log-rank statistical analysis (equivalent to Mantel–Haenszel test, two-tailed analysis), ns: not significant.
